# Supplementary material for: Effects of temperature and salinity stress on DNA methylation in a highly invasive marine invertebrate, the colonial ascidian Didemnum vexillum
Source: PeerJ. 2018 Jun 25;6:e5003. doi: 10.7717/peerj.5003 (PMC6022722; doi:10.7717/peerj.5003)
Supplement: Supplemental Information 1 [file peerj-06-5003-s001.docx]

**Table S1.** Analysis of variance (ANOVA) and degrees of freedom (d.f.) for colony growth rates (mean growth per day mm **±** 1 s.e.) at temperatures of 19°C, 25°C and 27°C, significant p-values are shown in bold.

| Source |  | d.f. | SS | MS | F value | *p* |
| --- | --- | --- | --- | --- | --- | --- |
| Temperature | | 2 | 5787 | 2893 | 7.82 | **0.0213** |
| Residuals |  | 6 | 2220 | 370 |  |  |

**Table S2.** Post hoc comparison of means between 27°C and 19°C, 25°C and 19°C, 27°C and 27°C using the Tukey’s test HSD

|  |  | Diff | lwr | upr | p adj |
| --- | --- | --- | --- | --- | --- |
| 27°C - 19°C | | -61.4301 | -109.621 | -13.2396 | **0.018486** |
| 25°C - 19°C | | -22.7651 | -70.9557 | 25.42539 | 0.376812 |
| 25°C - 27°C | | 38.665 | -9.52553 | 86.85553 | 0.107173 |

**Table S3.** Analysis of molecular variance (AMOVA) and degrees of freedom (d.f.) for methylation sensitive loci (MSL) in colonies held at three temperatures and sampled at three different time points: Time 0 (T0), Time 1 (T1) and Time 2 (T2). T0 shows baseline methylation prior to elevated temperature exposure, T1 shows methylation differences between temperature treatments following a gradual temperature increase of 1C per day until all treatment temperatures were reached, T2 shows methylation differences after three days of elevated temperature exposure. Significant p-values are shown in bold.

| Sampling times | Source | d.f. | SSD | MSD | Variance | Phi_ST | *p* value |
| --- | --- | --- | --- | --- | --- | --- | --- |
| T0 | Among groups | 2 | 72.69 | 36.34 | -2.011 | -0.0498 | 0.7093 |
|  | Within groups | 6 | 254.3 | 42.38 | 42.38 |  |  |
|  | Total | 8 | 326.9 | 40.87 |  |  |  |
| T1 | Among groups | 2 | 87.21 | 43.6 | 2.198 | 0.0561 | 0.2176 |
|  | Within groups | 6 | 222.1 | 37.01 | 37.01 |  |  |
|  | Total | 8 | 309.3 | 38.66 |  |  |  |
| T2 | Among groups | 2 | 129.6 | 64.81 | 7.798 | 0.1585 | **0.0215** |
|  | Within groups | 6 | 248.5 | 41.42 | 41.42 |  |  |
|  | Total | 8 | 378.1 | 47.27 |  |  |  |

**Table S4:** Analysis of molecular variance (AMOVA) and degrees of freedom (d.f.) for changes in methylation sensitive loci (MSL) over three sampling time points (T0, T1, T2) in colonies held at three temperatures (19°C, 25°C and 27°C). Significant p-values are shown in bold.

| Temperature (°C) | Source |  | d.f. | SSD | MSD | Variance | Phi_ST | *p* value |
| --- | --- | --- | --- | --- | --- | --- | --- | --- |
| 19 | Among groups |  | 2 | 91.21 | 45.6 | -1.01 | -0.0212 | 0.6019 |
|  | Within groups |  | 6 | 291.8 | 48.63 | 48.63 |  |  |
|  | Total |  | 8 | 383 | 47.88 |  |  |  |
| 25 | Among groups |  | 2 | 93.07 | 46.53 | -0.6572 | -0.0137 | 0.5637 |
|  | Within groups |  | 6 | 291 | 48.51 | 48.51 |  |  |
|  | Total |  | 8 | 384.1 | 48.01 |  |  |  |
| 27 | Among groups |  | 2 | 103.8 | 51.88 | 6.661 | 0.1727 | **0.0223** |
|  | Within groups |  | 6 | 191.4 | 31.9 | 31.9 |  |  |
|  | Total |  | 8 | 295.2 | 36.9 |  |  |  |

**Table S5.** Analysis of variance (ANOVA) and degrees of freedom (d.f.) for colony growth rates (mean growth per day mm **±** 1 s.e.) at 26, 28, 30, 32, and 34 PSU.

| Source |  | d.f. | SS | MS | F value | *p* value |
| --- | --- | --- | --- | --- | --- | --- |
| Salinity | | 4 | 253.9 | 63.48 | 2.066 | 0.161 |
| Residuals | | 10 | 307.3 | 30.73 |  |  |

**Table S6.** Analysis of molecular variance (AMOVA) and degrees of freedom (d.f.) for methylation sensitive loci (MSL) in colonies held at five salinities and sampled at three different time points: Time 0 (T0), Time 1 (T1) and Time 2 (T2). T0 shows baseline methylation prior to salinity treatment exposure, T1 shows methylation differences between salinity treatments following a gradual salinity decrease of one PSU per day until all salinity treatments were reached, T2 shows methylation differences after three days of salinity treatment exposure.

| Sampling times | Source | d.f. | SSD | MSD | Variance | Phi_ST | *p* value |
| --- | --- | --- | --- | --- | --- | --- | --- |
| T0 | Among groups | 4 | 103.5 | 25.88 | -1.445 | -0.05023 | 0.686 |
|  | Within groups | 10 | 302.2 | 30.22 | 30.22 |  |  |
|  | Total | 14 | 405.7 | 28.98 |  |  |  |
| T1 | Among groups | 4 | 136.1 | 34.04 | 0.8639 | 0.02674 | 0.3518 |
|  | Within groups | 10 | 314.4 | 31.44 | 31.44 |  |  |
|  | Total | 14 | 450.6 | 32.19 |  |  |  |
| T2 | Among groups | 4 | 61.76 | 15.44 | -1.131 | -0.06389 | 0.8328 |
|  | Within groups | 10 | 188.3 | 18.83 | 18.83 |  |  |
|  | Total | 14 | 250.1 | 17.86 |  |  |  |

**Table 7.** Analysis of molecular variance (AMOVA) and degrees of freedom (d.f.) for changes in methylation sensitive loci (MSL) over three sampling time points (T0, T1, T2) in colonies held at five salinities (26, 28, 30, 32 and 34 PSU).

| Salinity (PSU) | Source | d.f. | SSD | MSD | Variance | Phi_ST | *p value* |
| --- | --- | --- | --- | --- | --- | --- | --- |
| 26 | Among groups | 2 | 14.51 | 7.254 | -3.474 | -0.2446 | 0.9902 |
|  | Within groups | 6 | 106.1 | 17.68 | 17.68 |  |  |
|  | Total | 8 | 120.6 | 15.07 |  |  |  |
| 28 | Among groups | 2 | 29.85 | 14.93 | -1.471 | -0.08232 | 0.857 |
|  | Within groups | 6 | 116 | 19.34 | 19.34 |  |  |
|  | Total | 8 | 145.9 | 18.24 |  |  |  |
| 30 | Among groups | 2 | 20.59 | 10.29 | -2.484 | -0.1628 | 0.9553 |
|  | Within groups | 6 | 106.5 | 17.75 | 17.75 |  |  |
|  | Total | 8 | 127.1 | 15.88 |  |  |  |
| 32 | Among groups | 2 | 42.27 | 21.13 | -1.544 | -0.06373 | 0.784 |
|  | Within groups | 6 | 154.6 | 25.76 | 25.76 |  |  |
|  | Total | 8 | 196.9 | 24.61 |  |  |  |
| 34 | Among groups | 2 | 73.37 | 36.68 | 4.803 | 0.1774 | 0.0567 |
|  | Within groups | 6 | 133.7 | 22.28 | 22.28 |  |  |
|  | Total | 8 | 207 | 25.88 |  |  |  |
